# Identifying Susceptibility Genes and Shared Genetic Architecture for Longevity and Muscle Weakness

**DOI:** 10.1002/jcsm.70197

**Published:** 2026-01-26

**Authors:** Yilong Lin, Yun Zhang, Shengjie Lin, Songsong Wang, Zhiqiang Que, Yue Zhang, Jing She, Ruidan Zhao, Jiawei Chen, Anqi Qiu, Shinan Wu, Ruiqin Yang, Liyi Zhang, Qingmo Yang

**Affiliations:** ^1^ Depeartment of Breast Surgery, the First Affiliated Hospital of Xiamen University School of Medicine, Xiamen University Xiamen China; ^2^ School of Medicine Xiamen University Xiamen China; ^3^ Medical College Guangxi University Nanning China; ^4^ Department of Hematology, Xiangya Hospital, Xiangya School of Medicine, Central South University Changsha China; ^5^ Institute of Health Informatics University College London London UK

**Keywords:** genome‐wide cross‐trait analysis, longevity, muscle weakness, sarcopenia, shared genetic architecture, transcriptome‐wide association studies

## Abstract

**Background:**

Longevity and muscle strength are heritable traits, and age‐related muscle weakness is a major contributor to disability in older adults. However, the susceptibility genes and shared genetic mechanisms underlying lifespan and sarcopenia remain unclear. This study aimed to identify genes associated with longevity and muscle weakness and to characterize their shared genetic architecture.

**Methods:**

We integrated the largest genome‐wide association studies (GWAS) on longevity (age > 90th: *n* = 11 262 cases; age > 99th: *n* = 3484 cases) and muscle weakness (European Working Group on Sarcopenia in Older People (EWGSOP): *n* = 48 596 cases; Foundation for the National Institutes of Health (FNIH): *n* = 20 335 cases) with Genotype‐Tissue Expression (GTEx) v8 multi‐tissue expression quantitative trait locus (eQTL) data. Gene–trait associations were evaluated using multi‐tissue and single‐tissue TWAS, and validated using Multi‐marker Analysis of GenoMic Annotation (MAGMA). Mendelian randomization (MR) and colocalization were applied to test causality and shared variants. Cross‐trait genetic correlation was estimated with LDSC, and pleiotropic loci were identified by pleiotropy analysis under the composite null hypothesis (PLACO) followed by Functional Mapping and Annotation (FUMA)/MAGMA annotation.

**Results:**

Across TWAS approaches, APOC1 and TOMM40 were identified as longevity‐associated genes, while DYM and TGFA were susceptibility genes for muscle weakness. In MR analysis, higher expression of APOC1 and TOMM40 increased the odds of longevity (OR > 1, *p* < 0.05), whereas higher expression of DYM and TGFA reduced the risk of muscle weakness (OR < 1, *p* < 0.05). Colocalization supported shared causal variants for APOC1 (rs429358, PP.H4 = 0.81) and TOMM40 (rs429358, PP.H4 = 0.85) with longevity (age > 90th survival percentile), and for DYM and TGFA with muscle weakness defined by both EWGSOP and FNIH (PP.H4 > 0.80). A significant negative genetic correlation was observed between longevity and muscle weakness (Rg < 0, *p* < 0.05). Cross‐trait pleiotropy analysis identified several pleiotropic genes (PVRL2, PPP1R9A, SLC39A8 and the TOMM40/APOE/APOC1 gene cluster) that influence both longevity and muscle weakness.

**Conclusions:**

We identified susceptibility genes for longevity (APOC1, TOMM40) and muscle weakness (DYM, TGFA) and uncovered shared pleiotropic loci linking aging and muscle decline. These findings improve the understanding of the genetic architecture underlying aging‐related phenotypes and provide potential molecular targets for promoting healthy aging and reducing late‐life disability.

**Abbreviations**
eQTL: Expression quantitative trait locus
FDR: False discovery rate
FUMA: Functional Mapping and Annotation
GBJ: Generalized Berk–Jones test
GTEx: Genotype‐Tissue Expression
GWAS: Genome‐wide association study
LD: Linkage disequilibrium
LDSC: Linkage disequilibrium score regression
MAGMA: Multi‐marker Analysis of GenoMic Annotation
MR: Mendelian randomization
PLACO: Pleiotropy Adaptive Combination of Observations
SNP: Single‐nucleotide polymorphism
TWAS: Transcriptome‐wide association study
UTMOST: Unified Test for Molecular Signatures

## 1 Introduction

In recent decades, average life expectancy has demonstrated sustained growth consistently [1]. Longevity is a complex trait shaped by genetic and environmental factors and their interactions [1]. Previous twin studies have indicated that the heritability of longevity is approximately 20% to 30% (Supporting Information S1: References S1 and S2). For individuals who live beyond 85 years, this genetic contribution increases to 40% [3] (Supporting Information S1: Reference S3). Despite past genome‐wide association studies (GWAS) identifying genetic variations associated with longevity, the number of risk loci and longevity genes identified remains limited [3, 4] (Supporting Information S1: References S4–S7). Notably, only two loci, Apolipoprotein E (APOE) and GPR78, have demonstrated genome‐wide significance in multiple independent GWAS meta‐analyses [4, 5]. Specifically, the rs429358 variant (ApoE ε4) and rs7676745 (GPR78) were associated with a lower likelihood of longevity, whereas the rs7412 variant (ApoE ε2) showed the opposite [4, 5]. Nevertheless, the currently identified genetic variations account for only a portion of the heritability of longevity, indicating the need for additional methods to identify genes associated with this trait.

In addition to the genetics of longevity, age‐related diseases and their correlation with longevity have garnered significant attention [6]. Sarcopenia, a progressive and generalized skeletal muscle disorder characterized by the loss of muscle mass and function with age, is considered one of the most prevalent and complex muscle disorders [7] (Supporting Information S1: References S8 and S9). According to the European Working Group on Sarcopenia in Older People (EWGSOP), 4.6% of men and 7.9% of women, with an average age of 67, were affected by sarcopenia [8]. The changes in muscle mass and strength associated with sarcopenia are intrinsic outcomes of the human aging process [9] (Supporting Information S1: Reference S9) and are linked to an increased risk of various adverse conditions, including mobility impairment, higher morbidity and mortality rates [10] (Supporting Information S1: Reference S8 and S10). The causes and contributing factors of muscle weakness in later life remain to be fully elucidated (Supporting Information S1: Reference S9), and currently, there are no effective treatments to reverse or alter the progression of sarcopenia [11]. Given the heritable nature of muscle strength 52% (95% confidence interval (CI), 48%–55%) [11], further genetic research into the susceptibility genes for muscle weakness could facilitate the discovery of its underlying molecular mechanisms and potential drug targets.

Transcriptome‐wide association studies (TWAS) integrate expression quantitative trait loci with GWAS summary‐level data, facilitating the accurate identification of genes associated with complex disease risk and enabling investigation into gene–trait associations [12]. These genetic effects are mediated through the transcriptional activity regulated by genes (Supporting Information S1: Reference S11). Recent research has demonstrated the prevalence of expression quantitative trait locus (eQTL) sharing across different tissues [13] (Supporting Information S1: References S12 and S13). This suggests that multi‐tissue analysis, which integrates characteristics from various tissues, could enhance the accuracy of predictive modelling. Researchers have developed linkage disequilibrium score regression (LDSC) method to investigate the genetic correlation between two phenotypes (Supporting Information S1: Reference S14). Cross‐trait analyses utilizing the correlation of GWAS signals were used to identify pleiotropic loci, which can serve as potential intervention targets for simultaneously preventing and promoting these traits [14] (Supporting Information S1: References S15 and S16). A new method called ‘pleiotropy analysis under the composite null hypothesis (PLACO)’ has been developed, which used aggregated GWAS summary data based on a level‐α intersection–union test (IUT) to identify genetic variants that simultaneously affected the risk of two traits or diseases [15]. The PLACO method also accounted for GWAS correlations arising from shared controls between traits, significantly enhancing power compared to other methods [15].

Although both longevity and muscle function are highly heritable traits, the shared genetic mechanisms linking them remain largely unclear. Previous studies have mainly focused on either aging or sarcopenia individually, leaving the pleiotropic genetic factors that jointly influence lifespan and muscle weakness insufficiently characterized. To address this gap, we first identified susceptibility genes associated with longevity and sarcopenia separately using TWAS across multiple tissues, Mendelian randomization (MR) and colocalization analyses. We then performed cross‐trait genetic analyses to pinpoint pleiotropic genes influencing both traits, followed by functional mapping and gene‐based analyses to uncover the biological pathways underlying their shared architecture. This integrative framework provides novel insights into the common genetic basis of healthy aging and age‐related muscle decline, offering potential molecular targets for promoting longevity and mitigating sarcopenia.

## 2 Methods

**FIGURE 1:**
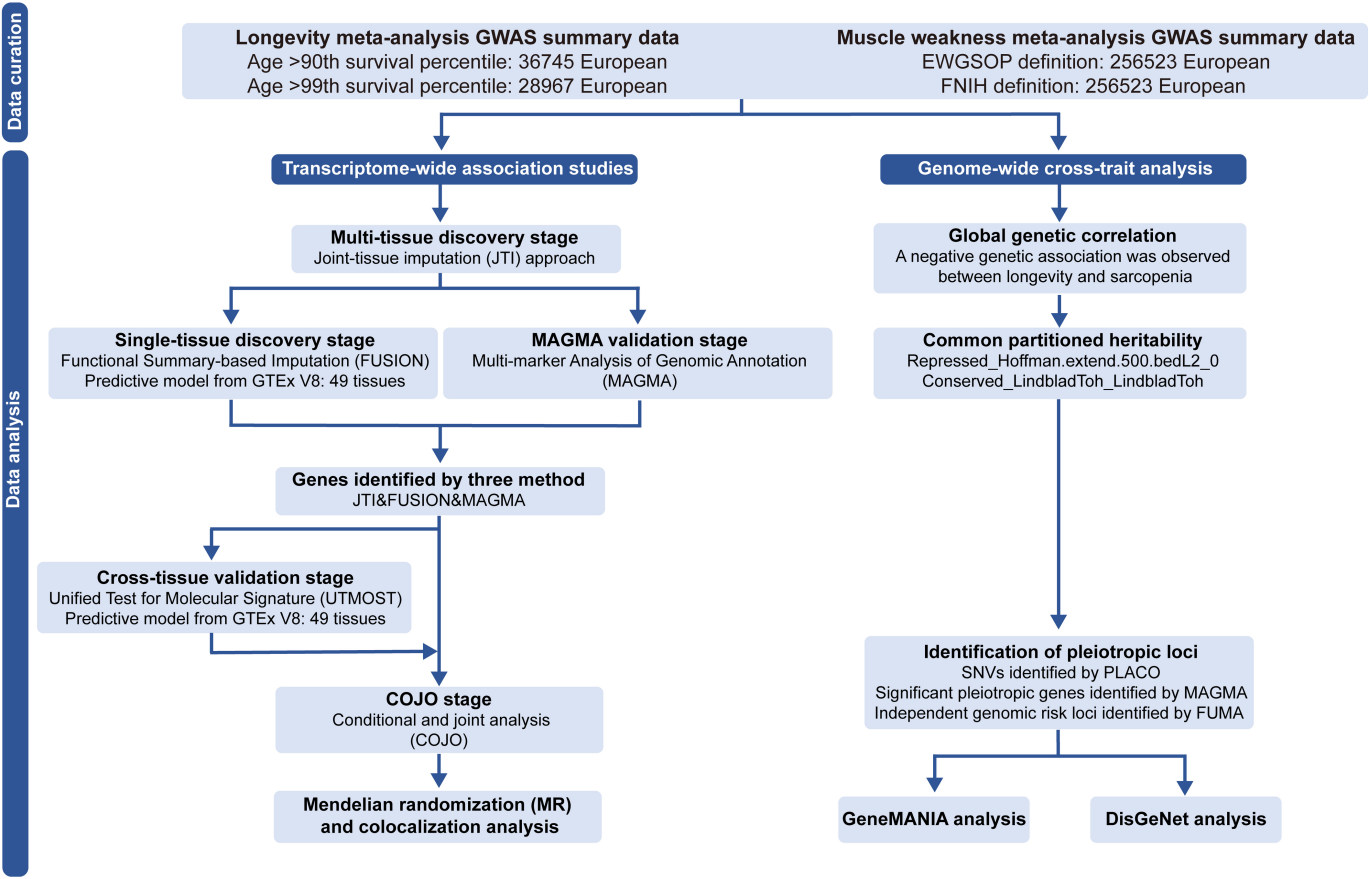
The flowchart of this study. GTEx, Genotype‐Tissue Expression; JTI, Joint‐tissue imputation; FUSION, Functional Summary‐based Imputation; MAGMA, Multi‐marker Analysis of GenoMic Annotation; UTMOST, Unified Test for Molecular Signatures; COJO, Conditional and joint; MR, Mendelian Randomization; GWAS, genome‐wide association studies; PLACO, pleiotropy analysis under the composite null hypothesis; FUMA, Functional Mapping and Annotation.

The flowchart of this study was illustrated schematically in Figure 1. In this study, we conducted a multi‐tissue TWAS analysis by integrating the largest meta‐analysis GWAS on longevity and sarcopenia with eQTL data from the Genotype‐Tissue Expression (GTEx) project v8. Then, we used Functional Summary‐based Imputation (FUSION) to assess associations within each tissue and validated these findings with Multi‐marker Analysis of GenoMic Annotation (MAGMA). MR and colocalization analyses were performed on candidate genes to clarify potential causal relationships and shared genetic variations. We explored the correlations between longevity and sarcopenia using S‐LDSC and conducted Functional Mapping and Annotation (FUMA) and MAGMA analyses based on PLACO results to further elucidate the possible shared genetic architecture of longevity and muscle weakness, providing insights for future comorbidity treatments.

### 2.1 Data Source of Longevity

The GWAS data source of longevity was obtained from a meta‐analysis of longevity GWAS [4], which included participants from 20 cohorts from populations of European, East Asian or African American ancestry. The individuals surviving at or beyond the age corresponding to the 90th/99th survival percentile were considered as the longevity cases (11 262/3484 cases), while the individuals who died at or before the age at the 60th percentile were identified as the control ones (25 483 controls).

### 2.2 Data Source of Muscle Weakness

The European Working Group on Sarcopenia in Older People 2 (EWGSOP2) defines sarcopenia (ICD‐10‐CM: M62.84) as initially identified by low muscle strength and confirmed by low muscle quantity or quality (Supporting Information S1: Reference S9). In this study, we selected meta‐analysis of low hand grip strength GWAS summary data for further analysis [16], which included two poor hand grip strength definitions: the EWGSOP definition (male: < 30 kg; female: < 20 kg) and the Foundation for the National Institutes of Health (FNIH) definition (male: < 26 kg; female: < 16 kg). Among 256 523 individuals aged 60 years or older from 22 cohorts, a total of 48 596 (18.9%) and 20 335 (7.9%) were identified as low grip strength based on EWGSOP and FNIH definitions [16].

### 2.3 Data Source of eQTL Files

The GTEx V8 consortium provides comprehensive transcriptomic profiles spanning 49 distinct anatomical sites, derived from postmortem human donors (*n* = 838) [17]. The number of samples in each of these tissues varied from 73 in the renal cortex to 706 in the skeletal muscle.

### 2.4 Multi‐Tissue TWAS Analyses

The Joint‐tissue imputation (JTI) method enhances the prediction accuracy of gene expression within individual tissues by leveraging shared genetic regulation across tissues (Supporting Information S1: Reference S17). In the JTI analysis, we integrated eQTL data from 49 related GTEx tissues using precomputed weights based on genetic variation covariance. To minimize false positives, false discovery rate (FDR) correction was applied to identify statistically significant TWAS genes from the JTI results. The JTI analysis was conducted using the SPrediXcan.py available on GitHub (https://github.com/hakyimlab/MetaXcan).

### 2.5 Single Tissue TWAS Analyses

To assess the gene–trait associations for both longevity and muscle weakness, we utilized the FUSION tool to conduct TWAS by integrating corresponding GWAS with eQTL data from 49 tissues in the GTEx V8 dataset (Supporting Information S1: Reference S18). Genes reaching the criteria (FDR < 0.05) were considered significantly associated with longevity and muscle weakness. Comprehensive analytical descriptions can be found in Supporting Information S2: Section S1.1.

### 2.6 Gene Analysis

To quantify gene's association with longevity and muscle weakness by aggregating SNP‐level association statistics into gene scores, we employed the MAGMA software (version 1.08) (https://cncr.nl/research/magma/) using its default parameters for further analysis (Supporting Information S1: References S19–S21).

### 2.7 Cross‐Tissue TWAS Analyses

To evaluate the comprehensive relationship between genes and traits at the organismal level, we employed Unified Test for Molecular Signatures (UTMOST) analyses (https://github.com/Joker‐Jerome/UTMOST?tab=readme‐ov‐file) across multiple tissues. UTMOST facilitated the identification of a more extensive array of genes within tissues that demonstrated both augmented trait heritability and enhanced imputation accuracy (Supporting Information S1: References S22 and 23). Subsequently, the Generalized Berk–Jones (GBJ) test was employed to amalgamate gene–trait associations by capitalizing on the covariance structure derived from single‐tissue statistics (Supporting Information S1: References S22 and S24). To ensure the robustness of our findings, associations were considered statistically significant only if they met the FDR threshold of less than 0.05.

### 2.8 Conditional and Joint (COJO) Analysis

For a locus with multiple associated traits, we used COJO analysis, a post‐processing module in FUSION, to identify conditionally independent genes at each locus (Supporting Information S1: Reference S18). COJO analysis enhances the understanding of the genetic structure underlying trait variation by accounting for linkage disequilibrium (LD) between markers (Supporting Information S1: Reference S25). Subsequent to this analysis, genes that manifest independent associations are considered jointly significant, whereas those that no longer demonstrate significance are classified as marginally significant.

### 2.9 MR

Utilizing candidate genes identified through TWAS analyses, we conducted an MR analysis with the ‘TwoSampleMR’ R package (version 0.6.8) to investigate the potential causal associations between these genes and longevity. Firstly, cis‐eQTLs specific to given tissues were extracted from GTEx V8. Cis‐eQTLs were deemed to meet the threshold for genome‐wide significance if they were located within 1 Mb on either side of the encoded gene. Subsequently, the SNPs that met the *p* value threshold (*p* < 1 × 10^−5^) were selected and subjected to LD clumping (*r* ^2^ < 0.001 and a 1 Mb distance). We selected cis‐eQTLs meeting the threshold of *p* < 1 × 10^−5^ because gene expression–based instruments are fewer in number and typically have weaker effects. Using a more stringent threshold (*p* < 5 × 10^−8^) would markedly reduce the number of available instrumental variables, thereby decreasing statistical power. Subsequently, the selected cis‐eQTLs were utilized as instrumental variables for the exposure in the present investigation. In the case of candidate genes with only one cis‐eQTL, the Wald ratio method was employed as the primary estimate. In the case of candidate genes comprising multiple SNPs, the primary analytical approach was the random‐effects inverse‐variance‐weighted (IVW) method. Because MR in this study was performed as a targeted validation step for a small number of TWAS‐identified candidate genes, a significance threshold of *p* < 0.05 was applied without FDR correction, following standard practice in focused causal inference analyses.

### 2.10 Colocalization Analysis

To determine whether the notable MR results were influenced by the overlap of causal variation loci, we employed colocalization analysis to prioritize candidate longevity and muscle weakness genes using the ‘coloc’ R package in R software (version 4.3.3) (Supporting Information S1: Reference S26). In the framework of colocalization, the posterior probability for hypothesis 4 (PP.H4) suggests two traits are influenced by sharing causal variants. In our analysis, a PP.H4 value exceeding 0.8 was considered statistically significant. Stacked regional plots were plotted to investigate the specific genomic regions in both GTEx V8 and interested traits.

### 2.11 Cross‐Trait Genetic Correlation

We conducted a genetic correlation analysis using LDSC analysis to estimate the genetic link between longevity and muscle weakness (Supporting Information S1: Reference S14). Genetic correlation represents the proportion of shared genetic variance between traits to the square root of the product of their heritability estimates (Supporting Information S1: Reference S27). Although polygenicity may inflate statistical estimates, LDSC can still provide robust and unbiased results under such conditions (Supporting Information S1: Reference S28). Further methodological details are available in Supporting Information S2: Section S1.2.

### 2.12 Partitioned Heritability

To further investigate the genetic correlation between longevity and sarcopenia, we conducted an S‐LDSC analysis on various genomic functional elements. This method classifies SNPs into functional groups and calculates the LD score for each group to estimate the genetic correlation within each category (Supporting Information S1: Reference S29). By estimating the genetic correlation of over 30 functional components, we elucidated the contributions of different elements to the overall genetic correlation between longevity and sarcopenia.

### 2.13 Identification of Pleiotropic Loci

For significant genetic associations in both unconstrained and constrained intercept LDSC, PLACO analysis has been employed to identify pleiotropic loci associated with complex traits [18]. Pleiotropic variants were considered significant at *p* < 5 × 10^−8^. Based on PLACO results, the FUMA platform was used to map potential pleiotropic loci and to identify candidate genes through MAGMA gene‐based analysis (Supporting Information S1: Reference S30). Detailed analytical procedures were provided in Supporting Information S2: Section S1.3.

### 2.14 GeneMANIA and DisGeNet

Potential interaction gene networks of susceptibility genes and pleiotropic genes were constructed using the GeneMANIA platform (https://genemania.org/). The GeneMANIA platform utilizes extensive functional association data to identify genes related to a given input gene (Supporting Information S1: Reference S31). The data encompasses protein and gene interactions, pathways, co‐expression, co‐localization and protein domain similarity. Through this approach, we can elucidate further insights into potential pathways associated with susceptibility genes and pleiotropic genes. DisGeNet analysis was conducted to identify the related disease and traits of pleiotropic genes through the Metascape database (https://www.metascape.org). DisGeNet is a comprehensive platform that integrates and standardizes data on disease‐associated genes and variants from multiple sources. It encompasses the full spectrum of human diseases, as well as normal and abnormal traits. The database includes information on over 24 000 diseases and traits, 17 000 genes and 117 000 genomic variants. This extensive repository provides a foundation for a range of applications in the fields of genomic medicine and drug research and development.

## 3 Results

### 3.1 TWAS Analyses in Multi‐Tissue and Single Tissue

In the multi‐tissue TWAS analyses, a total of 29, 18, 444 and 52 genes with FDR < 0.05 in at least one tissue were identified for longevity (age > 90/99th survival percentile) (Tables S1 and S2) and muscle weakness defined by EWGSOP/FNIH (Tables S3 and S4). For the validation of the single‐tissue TWAS analysis, a total of 71, 25, 642 and 75 genes with FDR < 0.05 in at least one tissue were identified for longevity (age > 90/99th survival percentile) (Tables S5 and S6) and muscle weakness defined by EWGSOP/FNIH (Tables S7 and S8). After multi‐tissue and single tissue TWAS analyses, 10 candidate genes of longevity (age > 90th survival percentile) satisfied the double strict thresholds, and these genes were all coding protein genes (APOC1, APOC4, BCAM, BLOC1S1, CEP89, LRRC37A2, MMP19, NECTIN2, TOMM40 and UBE2I) (Table S9). Among these genes, 5 genes (APOC1, APOC4, BCAM, TOMM40) were also identified as candidate genes of longevity (age > 99th survival percentile) (Table S10). In addition to these genes, cyclin‐dependent kinases (CDK) family (CDKN2A/2B/2B‐AS1) were also identified as candidate genes. JTI and FUSION identified 161 genes of muscle weakness (EWGSOP), comprising 153 coding protein genes and 8 non‐coding protein genes (DIO2‐AS1, HOXB‐AS2, LINC01831, MAPT‐AS1, MIR124‐2HG, MIR3936, SBF2‐AS1 and SNHG19) (Table S11). For muscle weakness defined by FNIH, 19 candidate genes were identified through JTI and FUSION approaches (Table S12).

### 3.2 Gene Analysis

MAGMA gene‐based test identified 4 (APOC1, TOMM40, APOE and PVRL2), 6 (APOE, APOC1, TOMM40, PVRL2, CAMKMT and KIF13B), 170 and 5 (TGFA, HLA‐DRB5, DYM, HLA‐DQA1 and PBX2) significant genes associated with longevity (age > 90/99th survival percentile) (Tables S13 and S14) and muscle weakness defined by EWGSOP (Table S15) and FNIH (Table S16).

### 3.3 Cross‐Tissue TWAS Analyses

**FIGURE 2:**
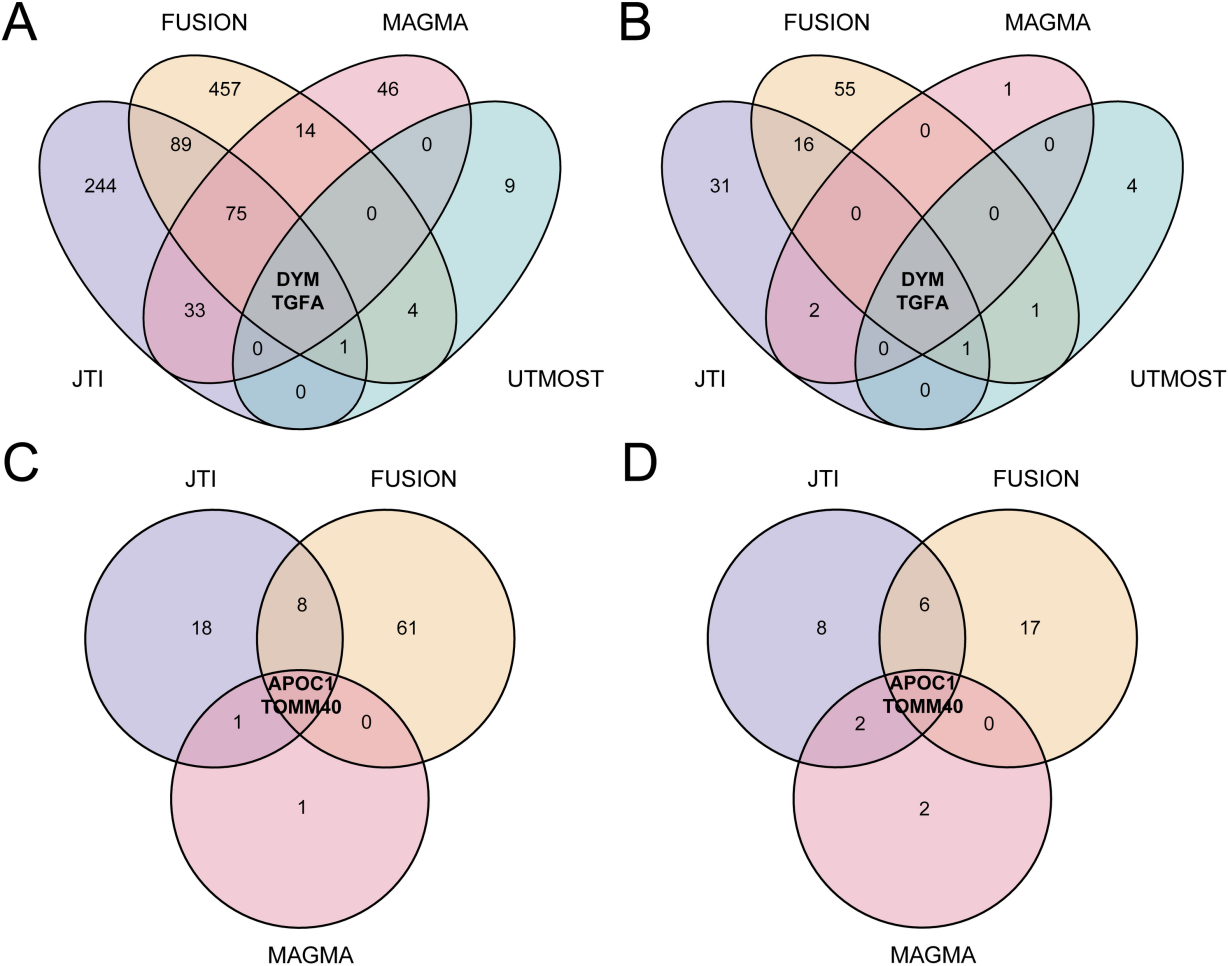
Venn diagrams of multi‐method Transcriptome‐wide association studies (TWAS) analyses for longevity and muscle weakness. (A) Venn diagrams of four TWAS methods for longevity (age > 90th survival percentile). (B) Venn diagrams of four TWAS methods for longevity (age > 99th survival percentile). (C) Venn diagrams of three TWAS methods for muscle weakness defined by European Working Group on Sarcopenia in Older People (EWGSOP). (D) Venn diagrams of three TWAS methods for muscle weakness defined by Foundation for the National Institutes of Health (FNIH). FUSION, Functional Summary‐based Imputation; MAGMA, Multi‐marker Analysis of GenoMic Annotation; JTI, Joint‐tissue imputation; UTMOST, Unified Test for Molecular Signatures.

UTMOST analyses were used to estimate the comprehensive association between genes and traits across multiple tissues. For longevity (age > 90th survival percentile), a total of 178 genes were identified (*p* < 0.05) (Table S17). After FDR correction of the data, only IGLJ3 showed statistically significant results (FDR < 0.05). For longevity (age > 99th survival percentile), IGLJ3 and IGLV1–44 were identified as candidate genes (FDR < 0.05) (Table S18). For muscle weakness defined by EWGSOP (Table S19) and FNIH (Table S20), UTMOST analyses identified 16 and 8 candidate genes, respectively (FDR < 0.05). At this stage, we integrated the significant results from multi‐tissue and single‐tissue TWAS analyses with genes identified through MAGMA and UTMOST evaluations. Cross‐tissue TWAS analysis was used as supplementary validation, provided the findings aligns with the first three TWAS methods. Finally, the combination of four TWAS methods revealed two common susceptibility genes (DYM and TGFA) for muscle weakness defined by both EWGSOP and FNIH criteria (Figure 2A,B). JTI, FUSION and MAGMA identified two promising longevity candidate genes (APOC1 and TOMM40) associated with survival beyond the 90th/99th percentiles of age (Figure 2C,D). However, these genes could not be validated in cross‐tissue TWAS analyses using the UTMOST method.

### 3.4 COJO Analysis

Longevity candidate genes (APOC1 and TOMM40) were located on chromosomes 19, and we conducted COJO analysis to avoid false positive results caused by LD (Table S21). In adipose subcutaneous, oesophageal mucosa, pituitary and suprapubic skin not exposed to sun, TOMM40 was a jointly significant gene for longevity (age > 90th survival percentile). In pituitary and suprapubic skin not exposed to sun, TOMM40 was a jointly significant gene for longevity (age > 99th survival percentile). Because of APOC1 significance solely in the oesophageal mucosa through the FUSION approach and the potential influence of LD, it was not further analysed. Since there were no other relevant genes within the window interval of the DYM gene (chromosome 18), COJO analysis was not conducted. The results of COJO analysis performing on the significant FUSION tissues of TGFA at chromosome 2 revealed that TGFA was a jointly significant gene in these tissues.

### 3.5 MR and Colocalization Analysis

**FIGURE 3:**
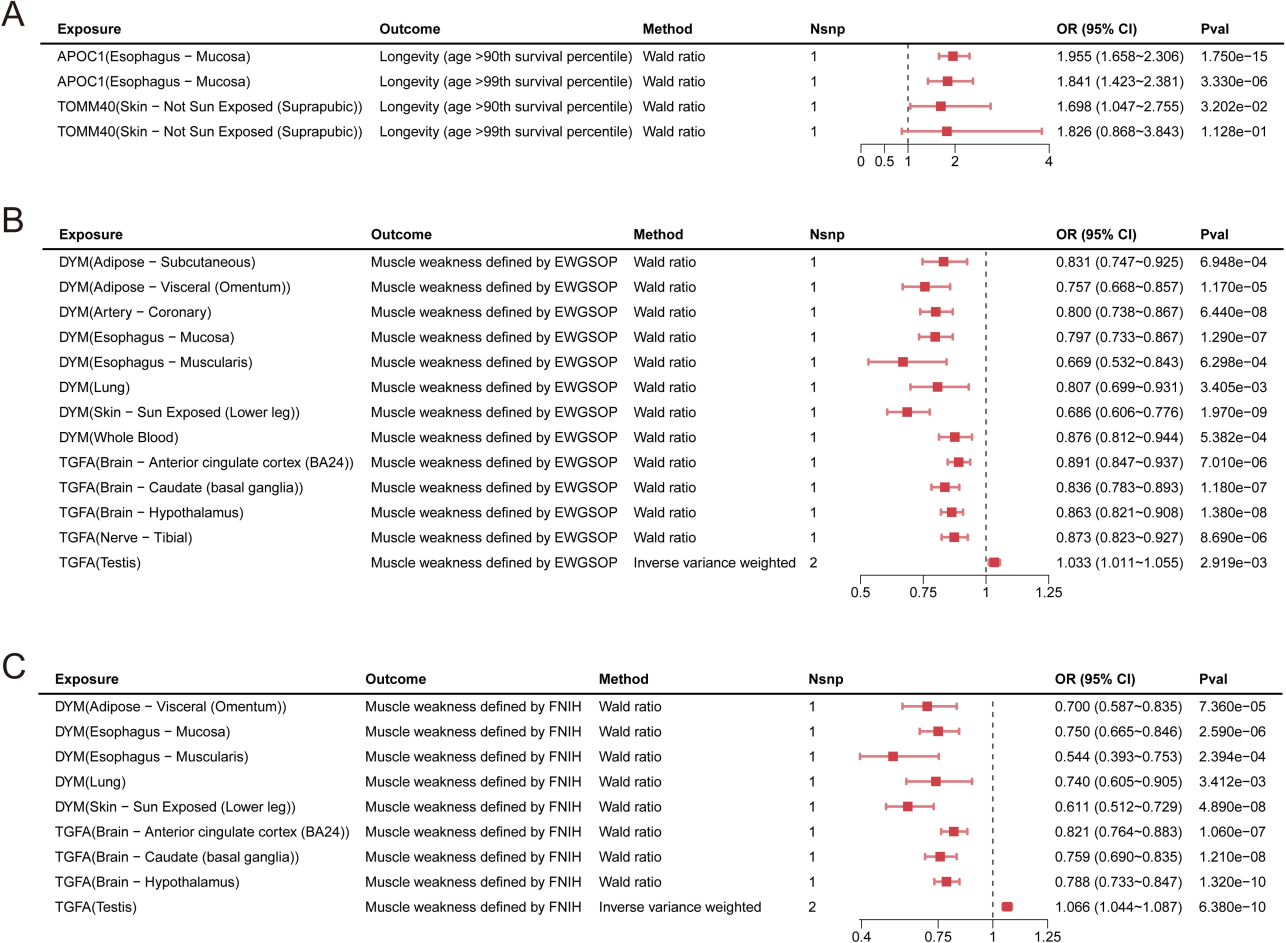
The causal associations between gene expression, longevity and muscle weakness identified by Mendelian randomization. (A) The causal associations between gene expression and longevity. (B) The causal association between gene expression and muscle weakness defined by European Working Group on Sarcopenia in Older People (EWGSOP). (C) The causal associations between gene expression and muscle weakness defined by Foundation for the National Institutes of Health (FNIH).

**FIGURE 4:**
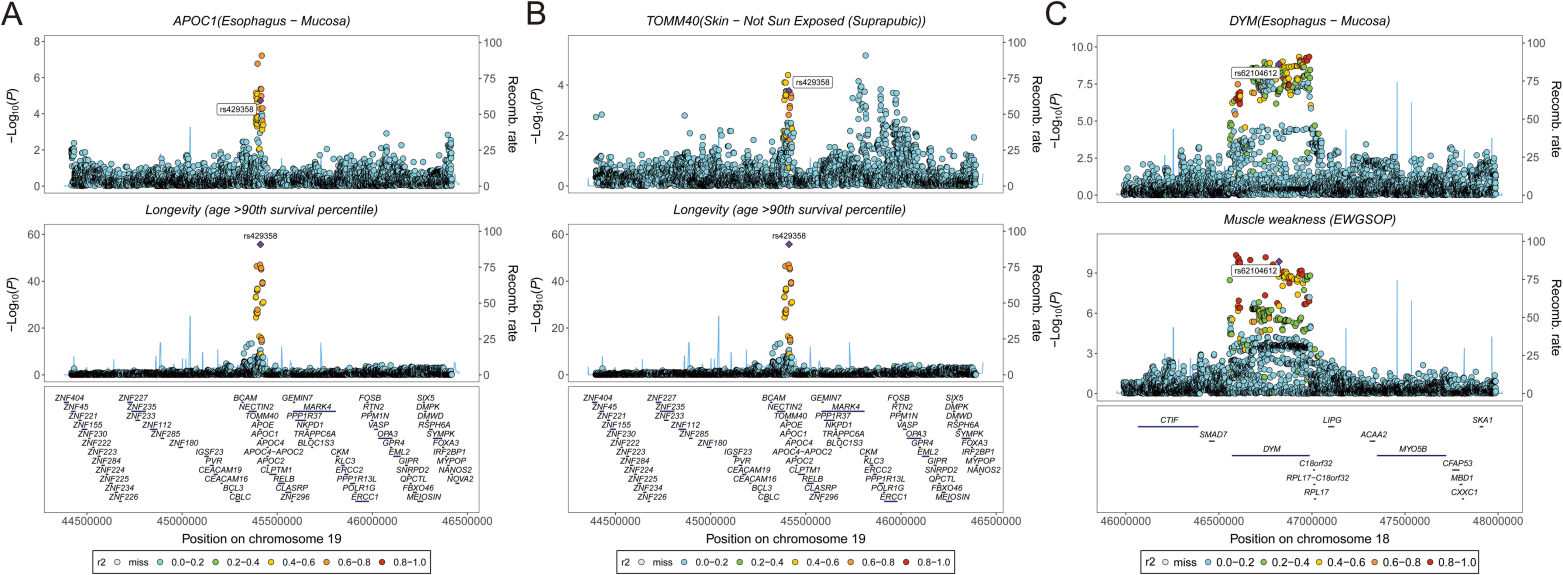
The results of colocalization analysis between candidate genes, longevity and muscle weakness. (A) APCO1 (oesophagus‐mucosa) and longevity (age > 90th survival percentile). (B) TOMM40 (Skin‐not sun exposed (Suprapubic)) and longevity (age > 90th survival percentile). (B) DYM (oesophagus‐mucosa) and muscle weakness defined by European Working Group on Sarcopenia in Older People (EWGSOP).

Based on significant results of FUSION and strict variables filtering, MR results revealed that the gene expression of APOC1 (OR = 1.96 [95% CI 1.66, 2.31], *p* = 1.75 × 10^−15^) and TOMM40 (OR = 1.70 [95% CI 1.05, 2.76], *p* = 3.20 × 10^−2^) were associated with longevity (age > 90th survival percentile) significantly (Figure 3A). Colocalization analysis indicated that APOC1 (rs429358, PP.H4 = 0.81, Figure 4A) and TOMM40 (rs429358, PP.H4 = 0.85, Figure 4B) shared the same variant with longevity (age > 90th survival percentile). For longevity (age > 99th survival percentile), APOC1 expression still increased the possibility of longevity (OR = 1.96 [95% CI 1.66, 2.31], *p* = 1.75 × 10^−15^, Figure 3A). However, it did not satisfy colocalization analysis threshold (PP.H4 = 0.50). For muscle weakness, DYM and TGFA could reduce the risk of muscle weakness defined by both EWGSOP and FNIH (Figure 3B,C). DYM expression from oesophageal mucosa may share the same variant with muscle weakness by EWGSOP (PP.H4 = 0.81, Figure 4C). Addition to oesophageal mucosa tissue (PP.H4 = 0.89), DYM expression from sun‐exposed skin also met the colocalization threshold (PP.H4 = 0.94) and shared the same variant with muscle weakness defined by FNIH (Table S22). Due to the absence of the top marker of DYM (oesophageal mucosa and sun exposed lower leg) in the marker dataset, the stacked regional plot was not drawn. At the same time, TGFA expression from five brain regions (amygdala, anterior cingulate cortex (BA24), caudate (basal ganglia), frontal cortex (BA9) and hypothalamus) may share the same variant with muscle weakness defined by both EWGSOP and FNIH (PP.H4 > 0.80) (Table S22).

### 3.6 Cross‐Trait Genetic Correlation

The single trait LDSC analysis revealed GWAS‐based estimates of heritability for longevity and muscle weakness (GWAS90th = 0.087, GWAS99th = 0.098, GWAS_EWGSOP_ = 0.043 and GWAS_FNIH_ = 0.021), respectively. The unconstrained intercept LDSC analysis indicated negative genome‐wide correlations between longevity and muscle weakness (Table S23). Then the constrained intercept LDSC analysis was used as sensitivity analysis. The genetic associations remain significant, except for the genetic association between longevity (age > 99th survival percentile) and muscle weakness defined by EWGSOP (Table S23).

### 3.7 Partitioned Heritability

SNPs associated with longevity (age > 90th/99th survival percentile) exhibited enrichment in 13 and 5 of 53 functional categories (*p* < 0.05) (Tables S24 and S25). Three functional categories, incl7uding Repressed_Hoffman.extend.500.bedL2_0, TFBS_ENCODE.bedL2_0 and Conserved_LindbladToh.extend.500.bedL2_0, were significant in both longevity enrichment. The SNPs related to muscle weakness defined by EWGSOP showed enrichment in 31 of 53 functional categories (Table S26). Meanwhile, muscle weakness defined by the FNIH was enriched in 17 functional categories (Table S27). Notably, Repressed_Hoffman.extend.500.bedL2_0 and Conserved_LindbladToh_LindbladToh demonstrated significant associations with both longevity and muscle weakness, regardless of the definition used.

### 3.8 Shared Loci Between Longevity and Muscle Weakness

In the investigation of genetic associations between longevity (age > 90th survival percentile) and muscle weakness as characterized by the EWGSOP and FNIH criteria, a comprehensive PLACO analysis identified 14 and 51 SNPs with potential polymorphic variations, respectively (Tables S28 and S29, P.placo < 5 × 10^−8^). Further analysis using MAGMA and FUMA revealed five significant pleiotropic genes (PVRL2, APOE, TOMM40, APOC1 and SLC39A8) and two independent genomic risk loci (rs35225200 and rs10119), associated with longevity (age > 90th survival percentile) and muscle weakness defined by EWGSOP. Similarly, for longevity and muscle weakness defined by FNIH, the replicated analyses identified five pleiotropic genes (PVRL2, APOE, TOMM40, APOC1 and PPP1R9A) and two independent genomic risk loci (rs10953133 and rs10119). When examining the genetic association between longevity (age > 99th survival percentile) and muscle weakness according to FNIH criteria, PLACO analysis identified 17 SNPs with potential polymorphic variations (Table S30). MAGMA and FUMA analyses further identified two independent genomic risk loci (rs10953133 and rs10119) and four significant pleiotropic genes (PVRL2, APOE, TOMM40 and PPP1R9A) within this association. In summary, six genes (PVRL2, APOE, TOMM40, PPP1R9A, APOC1 and SLC39A8) were identified as pleiotropic genes of the association between longevity and muscle weakness.

### 3.9 GeneMANIA and DisGeNet Analysis

**FIGURE 5:**
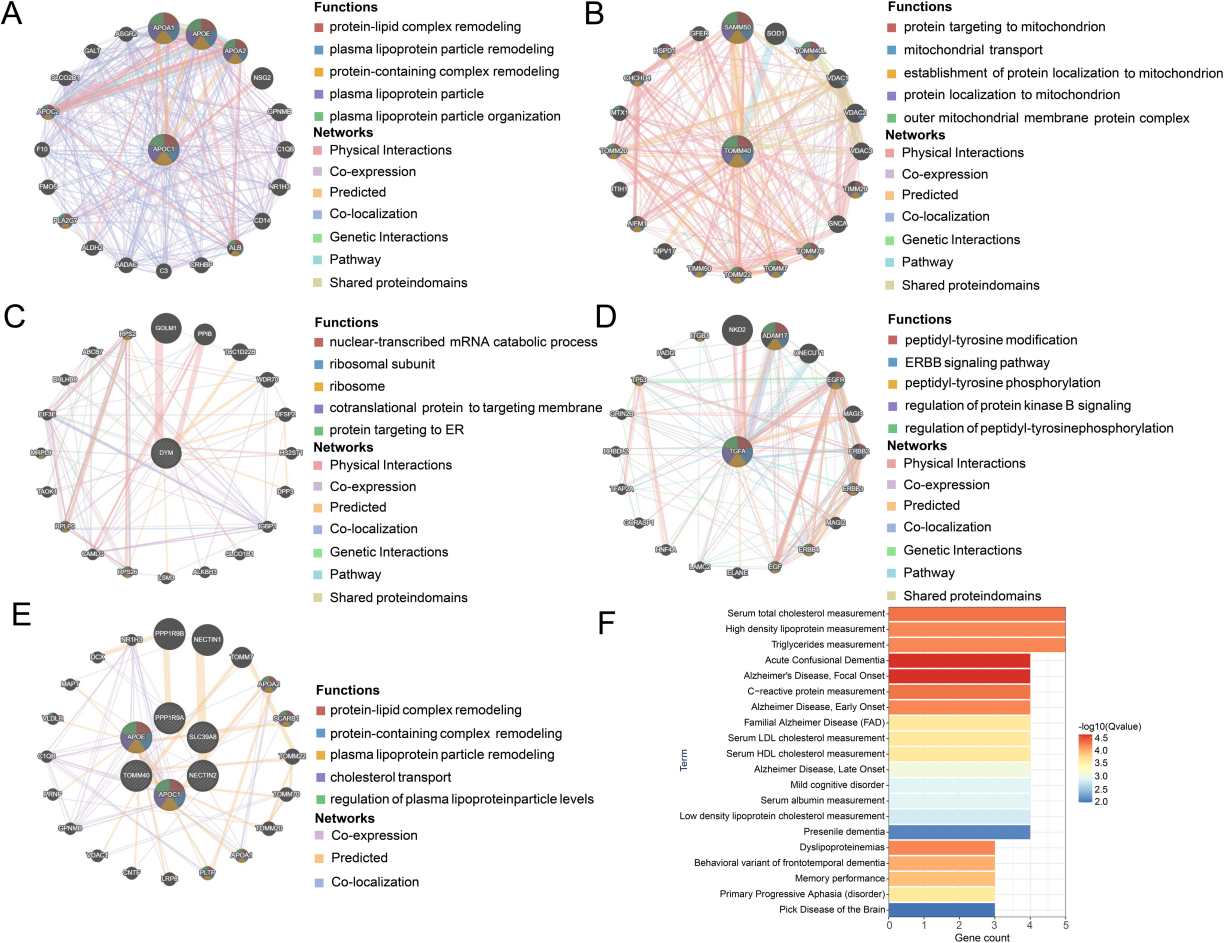
GeneMANIA networks centered on (A) APOC1, (B) TOMM40, (C) DYM, (D) TGFA, (E) six pleiotropic genes of the association between longevity and muscle weakness (PVRL2, APOE, TOMM40, PPP1R9A, APOC1 and SLC39A8). (F) The human diseases, normal and abnormal traits identified through DisGeNet analysis of six pleiotropic genes.

The potential interaction gene networks centered on longevity and muscle weakness susceptibility genes were illustrated in Figure 5. In the APOC1‐related gene network, the most significantly enriched functional pathways included protein‐lipid complex remodelling, plasma lipoprotein particle remodelling, protein‐containing complex remodelling, plasma lipoprotein particle and plasma lipoprotein particle organization (Figure 5A, Table S31). Meanwhile, the TOMM40‐related gene network was enriched in protein targeting to mitochondrion, mitochondrial transport, establishment of protein localization to mitochondrion, protein localization to mitochondrion and outer mitochondrial membrane protein complex (Figure 5B, Table S32). Only the nuclear‐transcribed mRNA catabolic process was significantly enriched in the DYM‐related gene network (Figure 5C, Table S33). The most critical functional pathways of the TGFA‐related gene network included peptidyl‐tyrosine modification, ERBB signalling pathway, peptidyl‐tyrosine phosphorylation, regulation of protein kinase B signalling and regulation of peptidyl‐tyrosine phosphorylation (Figure 5D, Table S34). GeneMANIA analysis revealed that protein‐lipid complex remodelling, protein‐containing complex remodelling, plasma lipoprotein particle remodelling, cholesterol transport and regulation of plasma lipoprotein particle levels were involved in the association between longevity and muscle weakness (Figure 5E, Table S35). The results of DisGeNet analysis indicated that these genes may be linked to nervous system diseases, including acute confusional dementia, Alzheimer's disease, frontotemporal dementia and cognitive disorder (Figure 5F, Table S36). C‐reactive protein, total cholesterol high density lipoprotein, triglycerides, albumin and low density lipoprotein cholesterol in serum may play an important role in this and may serve as biomarkers for early diagnosis (Figure 5F).

## 4 Discussion

Utilizing data from large‐scale GWAS and the GTEx V8 eQTL database, we systematically evaluated the genetic susceptibility of gene expression to longevity and muscle weakness risk. Through multiple TWAS and validation efforts, we identified two longevity‐associated genes (APOC1/TOMM40) and two muscle weakness‐associated genes (DYM/TGFA). MR and colocalization analyses further confirmed the causal relationships between these genes and their respective traits, along with shared genetic variations. Notably, we observed a strong genetic correlation between longevity and muscle weakness, and identified key bridging genes and loci that may underlie the relationship between these two traits.

Previous GWAS have identified multiple loci associated with human longevity, including variants near APOE, FOXO3 and CDKN2A/B [4] (Supporting Information S1: References S4 and S32). However, the susceptibility genes of these traits have not been thoroughly investigated using TWAS analysis. As the largest scale of GWAS about longevity, Deelen et al. found that rs7676745, located near GPR78, was associated with decreased longevity [4]. They also confirmed significant genetic associations at two loci previously implicated in longevity, FOXO3 and CDKN2A/B [4]. Two other longevity loci, rs6475609 (CDKN2B‐AS1) and rs2149954 (chromosome 5q33.3) were identified in previous studies (Supporting Information S1: References S4 and S32). These findings underscored the polygenic nature of longevity, involving multiple genes and pathways and contributed to further investigations into aging and longevity. Building upon these GWAS findings, our TWAS analyses identified APOC1 and TOMM40, which are located in the APOE region and represent novel longevity‐associated candidates, suggesting that transcriptomic regulation in this locus may mediate age‐related processes.

The TOMM40/APOE/APOC1 gene cluster on chromosome 19 has been extensively studied for its association with human longevity [19, 20] (Supporting Information S1: Reference S4). Through multimodal TWAS analysis and genome‐wide cross‐trait analysis, we confirmed the crucial role of APOC1 and TOMM40 in longevity and muscle weakness. The colocalization analyses identified the rs429358 variant as the top SNP, which was considered a variant related to ApoE ε4 [4]. The shorter lifespan of ApoE ε4 allele carriers is primarily due to cardiovascular and neurodegenerative diseases, but the underlying mechanisms remain unclear [21]. TOMM40 encodes the translocase of the outer mitochondrial membrane 40 (TOM40) protein, a crucial component of the mitochondrial protein import machinery [22]. Mitochondrial function is essential for cellular energy metabolism and homeostasis [23], and TOMM40 genetic variants are associated with healthy aging and longevity [24]. Linnertz et al. revealed that the TOMM40 variants could regulate the gene expression of both TOMM40 and APOE [25]. The interaction of TOMM40 with other genes may influence lipid metabolism [26]. A recent study indicated that TOMM40 regulates hepatic lipid metabolism via an LXR‐dependent mechanism. Its knockdown disrupts mitochondria and endoplasmic reticulum contact sites, leading to activation of LXRB‐mediated transcription of APOE and LDLR, which promotes VLDL uptake and lipid droplet accumulation (Supporting Information S1: Reference S33). APOC1 encodes a protein involved in lipid metabolism, modulating the clearance of triglyceride‐rich lipoproteins [27]. Its interaction with APOE affects lipid profiles, which are crucial for maintaining muscle mass and function. In the context of sarcopenia, fat infiltration into muscle tissue is a hallmark of both aging and muscle wasting [28]. Alterations in fatty acid metabolism, including the accumulation of intramuscular lipids, contribute to muscle insulin resistance and ceramide accumulation, exacerbating muscle degeneration [29]. Lipid‐related interventions have shown potential in improving human health span, and modulating lipid metabolism may ameliorate age‐related diseases and enhance longevity [30]. Taken together, these findings implicate lipid metabolic pathways as a potential link between TOMM40/APOE/APOC1 and ageing phenotypes, although this remains a hypothesis that requires further experimental confirmation.

In this study, DYM was identified as a susceptibility gene of muscle weakness. The DYM gene encodes dymeclin, a protein essential for normal skeletal development and brain function [31]. Mutations in DYM are linked to Dyggve–Melchior–Clausen syndrome and Smith–McCort dysplasia, both characterized by skeletal abnormalities. In a previous study, mice lacking DYM presented with endochondral bone formation similar to the syndrome, and Dym‐mutant cells display multiple defects in vesicle traffic related to Golgi markers [32]. Notably, dymeclin is highly expressed in human skeletal muscle [33], suggesting a potential role in muscle function. Given that DYM mutations lead to skeletal defects, it is plausible that dymeclin contributes to muscle integrity and function. Further research is warranted to elucidate dymeclin's role in muscle physiology and its potential involvement in muscle weakness.

Another susceptibility gene of muscle weakness, TGFA, encodes transforming growth factor alpha, which is suggested to be a well‐characterized growth factor and plays a key role in neural nutrition [34]. This growth factor has been shown to be upregulated during the acute injury response of motor neurons, where it promotes neuronal survival [35] (Supporting Information S1: Reference S34). However, the direct evidence linking TGFA to neurodegenerative diseases such as Amyotrophic Lateral Sclerosis or Parkinson's disease is currently limited. Our initial bioinformatics indicated that TGFA was associated with peptidyl‐related functions, which may contribute to growth factor secretion. The neurotrophic properties of TGFA are particularly relevant in the context of muscle weakness, as the survival and function of motor neurons are crucial for effective neuromuscular communication. In a large‐scale GWAS involving 656 individuals, the allele associated with increased grip strength at TGFA was found to be associated with a lower proportion of Type I (slow‐twitch oxidative) muscle fibres and a corresponding tendency toward a higher proportion of Type IIB (fast‐twitch glycolytic) fibres [36]. Recent studies have confirmed that the reduction in Type II fibre size and flattening were aging‐associated morphological features rather than structural characteristics universally present across all age groups of skeletal muscle. With advancing age, the cross‐sectional area of Type II and IIA fibres markedly decreases and becomes more flattened, whereas Type I fibres remain largely preserved (Supporting Information S1: Reference S35). These age‐specific alterations are accompanied by increased denervation and reduced satellite cell density, leading to impaired contractile performance and postural control in older adults (Supporting Information S1: Reference S36). The cell‐type specificity of TGFA expression within skeletal muscle lineages remains incompletely defined. However, existing evidence indicates that the MAPK/ERK signalling pathway can mediate the conversion of muscle fibres from fast‐twitch (Type II) to slow‐twitch (Type I) phenotypes (Supporting Information S1: Reference S37). The TGF‐*β*/Smad pathway plays a critical role in regulating satellite cell proliferation, differentiation and myofiber specification (Supporting Information S1: References S38 and 39). Based on these mechanisms, TGFA may serve as a potential dual‐action therapeutic target that promotes skeletal muscle regeneration and preserves neuromuscular stability. Although current evidence remains limited, future translational studies exploring TGFA‐mediated regulation of muscle fibre type conversion and neuromuscular junction integrity may offer new strategies for the prevention and treatment of sarcopenia, thereby strengthening the bridge between molecular mechanisms and clinical application.

A total of six potential pleiotropic genes (PVRL2, APOE, TOMM40, PPP1R9A, APOC1 and SLC39A8) were identified in this study. Among these genes, the TOMM40/APOE/APOC1 gene cluster was also considered as a susceptibility locus for longevity. Interestingly, PVRL2 is located in very close proximity to these genes, and emerging evidence suggests that it may be influenced by the genetic factors associated with APOE and TOMM40. Notably, Lu et al. have shown that the PVRL2‐TOMM40‐APOE region was associated with human longevity in a Han Chinese population [37], suggesting that this chromosomal region may represent a critical area linked to longevity, subject to both genetic and epigenetic influences. PVRL2 encodes a Type I membrane glycoprotein that contains two Ig‐like C2‐type domains and one Ig‐like V‐type domain, making it a key component of adhesive junctions in the plasma membrane. PVRL2 is critical for maintaining proper cell–cell adhesion and extracellular matrix (ECM) integrity [38], both of which are essential for tissue function and regeneration. Previous studies have highlighted the involvement of PVRL2 in neurodegenerative diseases like Alzheimer's disease [39] (Supporting Information S1: Reference S40) and in cancer [40] (Supporting Information S1: Reference S41), suggesting that it may play diverse roles across different pathophysiological contexts. However, whether PVRL2 acts as an independent or pleiotropic genetic locus influencing both longevity and muscle weakness remains a subject of ongoing research. The interaction between PVRL2 and the APOE‐TOMM40 region may be crucial in understanding the molecular mechanisms underlying muscle function and age‐related diseases, warranting further investigation into its potential as a therapeutic target in aging‐related muscle disorders.

Currently, multi‐omics association studies are common in identifying disease susceptibility genes. The largest GWAS meta‐analysis to date has consolidated multiple GWAS to identify several longevity genes and 15 susceptibility loci associated with muscle weakness. However, to date, there have been no TWAS studies about longevity and muscle weakness. This study employed TWAS analysis and cross‐trait association analysis to explore genes related to longevity and muscle weakness, focusing on PLACO to investigate potential loci or genes that share genetic variations between the two traits. Unlike traditional GWAS, which identify genetic variants without direct biological interpretation, TWAS incorporates gene expression data to pinpoint putative causal genes. Our use of multiple complementary approaches offers advantages beyond the classical single‐tissue TWAS implemented in FUSION. While FUSION captures tissue‐specific associations, JTI improves expression imputation accuracy by leveraging cross‐tissue regulatory sharing. UTMOST further increases statistical power by jointly modelling gene–trait associations across tissues using the GBJ framework. MAGMA provides an orthogonal gene‐based analysis independent of expression prediction models. By extending TWAS across multiple tissues, our method accounts for the diverse biological contexts in which genes may influence longevity and muscle weakness. The genome‐wide cross‐trait analysis quantifies genetic correlations and uncovers shared pathways, offering novel insights into the interconnected biology of these traits. Our study revealed overlapping pathways related to dementia, Alzheimer's disease and measurement of lipid‐related molecules, which were not fully captured in prior single‐trait analyses. The application of this integrative methodology highlighted its potential to enhance the resolution of genetic mapping and paved the way for future research on aging and age‐related diseases.

Our findings should be considered with several limitations. First, the study cohort's predominantly European ancestry limits the applicability of the results to other populations. Future validation studies should include more diverse demographic groups to assess the cross‐ethnic relevance of our findings. Second, the analyses were based primarily on publicly available GWAS and eQTL summary statistics, which may not fully capture rare variants or context‐specific gene regulatory effects that influence longevity and muscle weakness. Longitudinal and population‐based datasets will be valuable for validating temporal and causal relationships. While we employed multiple rigorous statistical and bioinformatic approaches to minimize the risk of false positives, further validation through comparative analyses of gene expression or sequencing results in relevant tissues would strengthen our conclusions. Importantly, the biological interpretations proposed for the TOMM40/APOE/APOC1 locus are based on genetic association patterns and known functional annotations, and should be viewed as hypothesis‐generating rather than definitive mechanistic evidence. The potential roles of lipid remodelling and mitochondrial protein import pathways in linking this locus to aging and muscle function remain to be experimentally confirmed through targeted in vitro and in vivo studies. Future work should also integrate single‐cell transcriptomic, proteomic and metabolomic data to dissect cell type–specific mechanisms and validate causal pathways across tissues. Despite these constraints, our work provides a foundational framework for understanding the genetic architecture of longevity and muscle weakness, offering insights that may inform future translational research into age‐related pathologies.

## 5 Conclusion

In conclusion, our study integrated multiple TWAS methods to identify two longevity genes (APOC1 and TOMM40) and two muscle weakness‐related genes (DYM and TGFA). We further observed a genetic interplay between prolonged lifespan and musculoskeletal decline, supported by shared pleiotropic loci. These findings enhance the current understanding of the overlapping genetic architecture underlying ageing‐related phenotypes. While these results suggest potential biological pathways that may influence both longevity and muscle strength, the functional mechanisms and translational relevance of these loci require further validation in experimental and clinical settings. This work provides a foundation for future research aimed at promoting healthy ageing and reducing late‐life disability.

## Funding

This study received funding from Fujian Provincial Natural Science Foundation of China (Grant No. 2024 J08318), the Xiamen Health and Wellness High‐Quality Development Science and Technology Program (Grant No. 2024GZL‐GG59), the First Affiliated Hospital of Xiamen University for Excellent Nurturing Program, China (XYP2023004), the Talent Introduction Research Foundation of the First Affiliated Hospital of Xiamen University (XYJ2024002) and the Beijing Medical Award Foundation (Grant No. YXJL‐2020‐0941‐0746).

## Ethics Stetement

This study utilized publicly available de‐identified data from participant studies that were already approved by an ethical standards committee for human experimentation, eliminating the need for separate ethical approval.

## Conflicts of Interest

The authors declare no conflicts of interest.

## Supporting information


**Data S1:** Supplementary information 1.

**Data S2:** Supplementary information 2.

**Table S1:** The TWAS analyses results of longevity (age > 90th survival percentile) in multi‐tissue through joint‐tissue imputation.
**Table S2:** The TWAS analyses results of longevity (age > 99th survival percentile) in multi‐tissue through joint‐tissue imputation.
**Table S3:** The TWAS analyses results of muscle weakness (European Working Group on Sarcopenia in Older People) in multi‐tissue through joint‐tissue imputation.
**Table S4:** The TWAS analyses results of muscle weakness (Foundation for the National Institutes of Health) in multi‐tissue through joint‐tissue imputation.
**Table S5:** FUSION identified significant genes associated with longevity (age > 90th survival percentile).
**Table S6:** FUSION identified significant genes associated with longevity (age > 99th survival percentile).
**Table S7:** FUSION identified significant genes associated with muscle weakness (European Working Group on Sarcopenia in Older People).
**Table S8:** FUSION identified significant genes associated with muscle weakness (Foundation for the National Institutes of Health).
**Table S9:** JTI and FUSION identified 10 genes of longevity (age > 90th survival percentile).
**Table S10:** JTI and FUSION identified 8 genes of longevity (age > 99th survival percentile).
**Table S11:** JTI and FUSION identified 161 genes of muscle weakness (EWGSOP).
**Table S12:** JTI and FUSION identified 19 genes of muscle weakness (FNIH).
**Table S13:** MAGMA gene‐based test identified 4 significant genes associated with longevity (age > 90th survival percentile).
**Table S14:** MAGMA gene‐based test identified 6 significant genes associated with longevity (age > 99th survival percentile).
**Table S15:** MAGMA gene‐based test identified 170 significant genes associated with muscle weakness (EWGSOP).
**Table S16:** MAGMA gene‐based test identified 5 significant genes associated with muscle weakness (FNIH).
**Table S17:** The cross‐tissue TWAS analyses results of longevity (age > 90th survival percentile) through UTMOST.
**Table S18:** The cross‐tissue TWAS analyses results of longevity (age > 99th survival percentile) through UTMOST.
**Table S19:** The cross‐tissue TWAS analyses results of muscle weakness (EWGSOP) through UTMOST.
**Table S20:** The cross‐tissue TWAS analyses results of muscle weakness (FNIH) through UTMOST.
**Table S21:** The results of conditional and joint analysis.
**Table S22:** The significant colocalization results of muscle weakness (PP.H4 > 0.8).
**Table S23:** The cross‐trait genetic correlation of longevity and muscle weakness.
**Table S24:** The partitioned heritability of longevity (age > 90th survival percentile).
**Table S25:** The partitioned heritability of longevity (age > 99th survival percentile).
**Table S26:** The partitioned heritability of muscle weakness defined by EWGSOP.
**Table S27:** The partitioned heritability of muscle weakness defined by FNIH.
**Table S28:** The significant results of PLACO analysis between longevity (age > 90th survival percentile) and muscle weakness defined by EWGSOP.
**Table S29:** The significant results of PLACO analysis between longevity (age > 90th survival percentile) and muscle weakness defined by FNIH.
**Table S30:** The significant results of PLACO analysis between longevity (age > 99th survival percentile) and muscle weakness defined by FNIH.
**Table S31:** The functions of potential interaction gene networks centered on APOC1.
**Table S32:** The functions of potential interaction gene networks centered on TOMM40.
**Table S33:** The functions of potential interaction gene networks centered on DYM.
**Table S34:** The functions of potential interaction gene networks centered on TGFA.
**Table S35:** The functions of potential interaction gene networks centered on longevity and muscle weakness susceptibility genes.
**Table S36:** The results of DisGeNet of longevity and muscle weakness susceptibility genes.

## Data Availability

All data for this study were publicly available and details could be found in the supplementary material of this article.
