# Supplementary material for: Identifying Susceptibility Genes and Shared Genetic Architecture for Longevity and Muscle Weakness
Source: J Cachexia Sarcopenia Muscle. 2026 Jan 26;17(1):e70197. doi: 10.1002/jcsm.70197 (PMC12835543; doi:10.1002/jcsm.70197)
Supplement: Supplementary file 1 — Data S1: Supplementary information 1. [file JCSM-17-e70197-s002.docx]

**SUPPLEMENTAL REFERENCES**

1. Herskind AM, McGue M, Holm NV, Sørensen TI, Harvald B, Vaupel JW: The heritability of human longevity: a population-based study of 2872 Danish twin pairs born 1870-1900. *Hum Genet* 1996, 97(3):319-323.
2. Skytthe A, Pedersen NL, Kaprio J, Stazi MA, Hjelmborg JVB, Iachine I *et al.*: Longevity studies in GenomEUtwin. *Twin Res* 2003, 6(5):448-454.
3. van den Berg N, Beekman M, Smith KR, Janssens A, Slagboom PE: Historical demography and longevity genetics: Back to the future. *Ageing Res Rev* 2017, 38:28-39.
4. Deelen J, Beekman M, Uh H-W, Broer L, Ayers KL, Tan Q *et al.*: Genome-wide association meta-analysis of human longevity identifies a novel locus conferring survival beyond 90 years of age. *Hum Mol Genet* 2014, 23(16):4420-4432.
5. Joshi PK, Pirastu N, Kentistou KA, Fischer K, Hofer E, Schraut KE *et al.*: Genome-wide meta-analysis associates HLA-DQA1/DRB1 and LPA and lifestyle factors with human longevity. *Nat Commun* 2017, 8(1):910.
6. Sebastiani P, Solovieff N, Dewan AT, Walsh KM, Puca A, Hartley SW *et al.*: Genetic signatures of exceptional longevity in humans. *PLoS One* 2012, 7(1):e29848.
7. Zeng Y, Nie C, Min J, Liu X, Li M, Chen H *et al.*: Novel loci and pathways significantly associated with longevity. *Sci Rep* 2016, 6:21243.
8. Polyzos SA, Mantzoros CS: Sarcopenia: still in relative definition-penia and severe treatment-penia. *Metabolism* 2024, 150:155717.
9. Cruz-Jentoft AJ, Bahat G, Bauer J, Boirie Y, Bruyère O, Cederholm T *et al.*: Sarcopenia: revised European consensus on definition and diagnosis. *Age Ageing* 2019, 48(1):16-31.
10. Yin J, Lu X, Qian Z, Xu W, Zhou X: New insights into the pathogenesis and treatment of sarcopenia in chronic heart failure. *Theranostics* 2019, 9(14):4019-4029.
11. Head ST, Dezem F, Todor A, Yang J, Plummer J, Gayther S *et al.*: Cis- and trans-eQTL TWASs of breast and ovarian cancer identify more than 100 susceptibility genes in the BCAC and OCAC consortia. *Am J Hum Genet* 2024, 111(6):1084-1099.
12. Battle A, Brown CD, Engelhardt BE, Montgomery SB: Genetic effects on gene expression across human tissues. *Nature* 2017, 550(7675):204-213.
13. Flutre T, Wen X, Pritchard J, Stephens M: A statistical framework for joint eQTL analysis in multiple tissues. *PLoS Genet* 2013, 9(5):e1003486.
14. Bulik-Sullivan B, Finucane HK, Anttila V, Gusev A, Day FR, Loh P-R *et al.*: An atlas of genetic correlations across human diseases and traits. *Nat Genet* 2015, 47(11):1236-1241.
15. Yu X-H, Yang Y-Q, Cao R-R, Cai M-K, Zhang L, Deng F-Y *et al.*: Rheumatoid arthritis and osteoporosis: shared genetic effect, pleiotropy and causality. *Hum Mol Genet* 2021, 30(21):1932-1940.
16. Lu H, Qiao J, Shao Z, Wang T, Huang S, Zeng P: A comprehensive gene-centric pleiotropic association analysis for 14 psychiatric disorders with GWAS summary statistics. *BMC Med* 2021, 19(1):314.
17. Zhou D, Jiang Y, Zhong X, Cox NJ, Liu C, Gamazon ER: A unified framework for joint-tissue transcriptome-wide association and Mendelian randomization analysis. *Nat Genet* 2020, 52(11):1239-1246.
18. Gusev A, Ko A, Shi H, Bhatia G, Chung W, Penninx BWJH *et al.*: Integrative approaches for large-scale transcriptome-wide association studies. *Nat Genet* 2016, 48(3):245-252.
19. de Leeuw CA, Stringer S, Dekkers IA, Heskes T, Posthuma D: Conditional and interaction gene-set analysis reveals novel functional pathways for blood pressure. *Nat Commun* 2018, 9(1):3768.
20. de Leeuw CA, Neale BM, Heskes T, Posthuma D: The statistical properties of gene-set analysis. *Nat Rev Genet* 2016, 17(6):353-364.
21. de Leeuw CA, Mooij JM, Heskes T, Posthuma D: MAGMA: generalized gene-set analysis of GWAS data. *PLoS Comput Biol* 2015, 11(4):e1004219.
22. Hu Y, Li M, Lu Q, Weng H, Wang J, Zekavat SM *et al.*: A statistical framework for cross-tissue transcriptome-wide association analysis. *Nat Genet* 2019, 51(3):568-576.
23. Ni J, Wang P, Yin K-J, Yang X-K, Cen H, Sui C *et al.*: Novel insight into the aetiology of rheumatoid arthritis gained by a cross-tissue transcriptome-wide association study. *RMD Open* 2022, 8(2).
24. Sun R, Hui S, Bader GD, Lin X, Kraft P: Powerful gene set analysis in GWAS with the Generalized Berk-Jones statistic. *PLoS Genet* 2019, 15(3):e1007530.
25. Liao C, Laporte AD, Spiegelman D, Akçimen F, Joober R, Dion PA *et al.*: Transcriptome-wide association study of attention deficit hyperactivity disorder identifies associated genes and phenotypes. *Nat Commun* 2019, 10(1):4450.
26. Giambartolomei C, Vukcevic D, Schadt EE, Franke L, Hingorani AD, Wallace C *et al.*: Bayesian test for colocalisation between pairs of genetic association studies using summary statistics. *PLoS Genet* 2014, 10(5):e1004383.
27. Long P, Tan H, Chen B, Wang L, Quan R, Hu Z *et al.*: Dissecting the shared genetic architecture between anti-Müllerian hormone and age at menopause based on genome-wide association study. *Am J Obstet Gynecol* 2024, 231(6).
28. Chen M, Xu X, Wang F, Xu X: Investigating causality and shared genetic architecture between body mass index and cognitive function: a genome-wide cross-trait analysis and bi-directional Mendelian randomization study. *Front Aging Neurosci* 2024, 16:1466799.
29. Okamura K, Kawai T, Hata K, Nakabayashi K: Lists of HumanMethylation450 BeadChip probes with nucleotide-variant information obtained from the Phase 3 data of the 1000 Genomes Project. *Genom Data* 2016, 7:67-69.
30. Watanabe K, Taskesen E, van Bochoven A, Posthuma D: Functional mapping and annotation of genetic associations with FUMA. *Nat Commun* 2017, 8(1):1826.
31. Warde-Farley D, Donaldson SL, Comes O, Zuberi K, Badrawi R, Chao P *et al.*: The GeneMANIA prediction server: biological network integration for gene prioritization and predicting gene function. *Nucleic Acids Res* 2010, 38(Web Server issue):W214-W220.
32. Bae H, Gurinovich A, Karagiannis TT, Song Z, Leshchyk A, Li M *et al.*: A Genome-Wide Association Study of 2304 Extreme Longevity Cases Identifies Novel Longevity Variants. *Int J Mol Sci* 2022, 24(1).
33. Yang NV, Chao JY, Garton KA, Tran T, King SM, Orr J *et al.*: TOMM40 regulates hepatocellular and plasma lipid metabolism via an LXR-dependent pathway. *Mol Metab* 2024, 90:102056.
34. Lisovoski F, Blot S, Lacombe C, Bellier JP, Dreyfus PA, Junier MP: Transforming growth factor alpha expression as a response of murine motor neurons to axonal injury and mutation-induced degeneration. *J Neuropathol Exp Neurol* 1997, 56(5):459-471.
35. Lee C, Woods PC, Paluch AE, Miller MS: Effects of age on human skeletal muscle: a systematic review and meta-analysis of myosin heavy chain isoform protein expression, fiber size, and distribution. *Am J Physiol Cell Physiol* 2024, 327(6):C1400-C1415.
36. Horwath O, Moberg M, Edman S, Philp A, Apró W: Ageing leads to selective type II myofibre deterioration and denervation independent of reinnervative capacity in human skeletal muscle. *Exp Physiol* 2024, 110(2):277-292.
37. Boyer JG, Prasad V, Song T, Lee D, Fu X, Grimes KM *et al.*: ERK1/2 signaling induces skeletal muscle slow fiber-type switching and reduces muscular dystrophy disease severity. *JCI Insight* 2019, 5(10).
38. Burks TN, Cohn RD: Role of TGF-β signaling in inherited and acquired myopathies. *Skelet Muscle* 2011, 1(1):19.
39. Gallardo FS, Cruz-Soca M, Bock-Pereda A, Faundez-Contreras J, Gutiérrez-Rojas C, Gandin A *et al.*: Role of TGF-β/SMAD/YAP/TAZ signaling in skeletal muscle fibrosis. *Am J Physiol Cell Physiol* 2025, 328(3):C1015-C1028.
40. Takei N, Miyashita A, Tsukie T, Arai H, Asada T, Imagawa M *et al.*: Genetic association study on in and around the APOE in late-onset Alzheimer disease in Japanese. *Genomics* 2009, 93(5):441-448.
41. Stamm H, Klingler F, Grossjohann E-M, Muschhammer J, Vettorazzi E, Heuser M *et al.*: Immune checkpoints PVR and PVRL2 are prognostic markers in AML and their blockade represents a new therapeutic option. *Oncogene* 2018, 37(39):5269-5280.
