# Supplementary material for: Identifying Susceptibility Genes and Shared Genetic Architecture for Longevity and Muscle Weakness
Source: J Cachexia Sarcopenia Muscle. 2026 Jan 26;17(1):e70197. doi: 10.1002/jcsm.70197 (PMC12835543; doi:10.1002/jcsm.70197)
Supplement: Supplementary file 2 — Data S2: Supplementary information 2. [file JCSM-17-e70197-s003.docx]

**Supplementary** **Methods**

- 1. **Single tissue TWAS analyses**

To assess the association between each gene and longevity and muscle weakness, we utilized the FUSION tool to conduct TWAS by integrating longevity GWAS with eQTL data from 49 tissues in the GTEx V8 dataset (Supporting Information: Reference S18). Initially, we employed European samples from the 1,000 Genomes Project to estimate the linkage disequilibrium (LD) between the prediction model and each GWAS locus. FUSION then combined multiple predictive models, including BLUP, BSLMM, LASSO, Elastic Net, and the top one model, to evaluate the overall influence of SNPs on gene expression weights. The model with the highest predictive performance was then selected to ascertain the gene weights. Subsequently, we integrated the genetic effects of longevity and muscle weakness, represented by GWAS Z-scores, with these gene weights to perform the TWAS for longevity and muscle weakness. Genes reaching the criteria (FDR <0.05) were considered significantly associated with longevity and muscle weakness.

- 1. **Cross-trait genetic correlation**

We conducted a genetic correlation analysis using LDSC analysis to estimate the genetic link between longevity and muscle weakness (Supporting Information: Reference S14). Genetic correlation represents the proportion of shared genetic variance between traits to the square root of the product of their heritability estimates (Supporting Information: Reference S27). The LDSC analysis was performed based on the LD structure of the European reference panel from the 1000 Genomes Project (Supporting Information: Reference S14). Initially, we applied LDSC to calculate the heritability of individual traits. Subsequently, we employed bivariate LDSC with an unconstrained intercept to determine the strength of genetic correlation between the two traits. As a sensitivity analysis, LDSC with a constrained intercept was also conducted. The genome-wide genetic correlation quantifies the average shared genetic effects between two traits, unaffected by environmental confounding factors, with values ranging from -1 to 1. Moreover, even though polygenicity might lead to inflated statistical estimates, LDSC can still provide accurate estimates (Supporting Information: Reference S28).

- 1. **Identification of pleiotropic loci**

For significant genetic associations in both unconstrained and constrained intercept LDSC, PLACO analysis has been employed to identify pleiotropic loci associated with complex traits. In this process, we first scored all SNPs using squared Z-scores, and excluded those SNPs with Z^2^ > 80%. Given the potential correlation between the two traits, we calculated and incorporated the correlation matrix of Z-scores. The hypothesis of no pleiotropy was then tested using level-α cross-over-unit test method, which determined the final pleiotropy P-value. Pleiotropic variants are considered significant if their P-value < 5×10⁻⁸. Based on PLACO results, we employed the FUMA tool to identify potential pleiotropic loci (Supporting Information: Reference S30). In addition, MAGMA analysis was used to identify candidate pleiotropic genes within or overlapping pleiotropic loci through FUMA platform.
